# Ligand-Induced Biased Activation of GPCRs: Recent Advances and New Directions from In Silico Approaches

**DOI:** 10.3390/molecules30051047

**Published:** 2025-02-25

**Authors:** Shaima Hashem, Alexis Dougha, Pierre Tufféry

**Affiliations:** Unité de Biologie Fonctionnelle et Adaptative, INSERM ERL 1133, CNRS UMR 8251, Université Paris Cité, F-75013 Paris, France; shaima.hashem@u-paris.fr (S.H.); alexis.dougha@u-paris.fr (A.D.)

**Keywords:** GPCRs, biased signaling, in silico approaches, molecular dynamics, artificial intelligence, deep learning

## Abstract

G-protein coupled receptors (GPCRs) are the largest family of membrane proteins engaged in transducing signals from the extracellular environment into the cell. GPCR-biased signaling occurs when two different ligands, sharing the same binding site, induce distinct signaling pathways. This selective signaling offers significant potential for the design of safer and more effective drugs. Although its molecular mechanism remains elusive, big efforts are made to try to explain this mechanism using a wide range of methods. Recent advances in computational techniques and AI technology have introduced a variety of simulations and machine learning tools that facilitate the modeling of GPCR signal transmission and the analysis of ligand-induced biased signaling. In this review, we present the current state of in silico approaches to elucidate the structural mechanism of GPCR-biased signaling. This includes molecular dynamics simulations that capture the main interactions causing the bias. We also highlight the major contributions and impacts of transmembrane domains, loops, and mutations in mediating biased signaling. Moreover, we discuss the impact of machine learning models on bias prediction and diffusion-based generative AI to design biased ligands. Ultimately, this review addresses the future directions for studying the biased signaling problem through AI approaches.

## 1. Introduction

Corresponding to almost 4% of the human genes (800 of ~20,000) [1], G-protein coupled receptors (GPCRs) are the largest class of eukaryotic transmembrane receptors. They can recognize extremely diverse stimuli, including light, odorants, tastants, ions, neurotransmitters, hormones, peptides, and lipids [2]. They are involved in the modulation of numerous mammalian physiological processes and, consequently, in a large number of pathologies that have made them targets of choice for drug development. Nearly 400 GPCRs are prime targets for drug development (over ~3100), and ~34% of the approved drugs target GPCRs [3,4].

Historically identified as localized at the cell surface [5], functional GPCRs have since been detected on the membrane of intracellular compartments, including endosomes, the endoplasmic reticulum, the Golgi apparatus [6], the outer and inner nuclear membranes, and the outer membrane of the mitochondria [7]. GPCRs transduce information brought about by a ligand on one (outer) side of the membrane, the N-terminal side of the GPCR, into an appropriate response on the other side of the membrane (inner), the C-terminal extremity of the GPCR. The C-terminal extremity of functional GPCRs identified so far faces the cytosol except for the GPCRs of mitochondria, where it faces the intermembrane space, and the GPCRs located in the inner nuclear membrane, where it faces the nucleoplasm.

**Figure 1:**
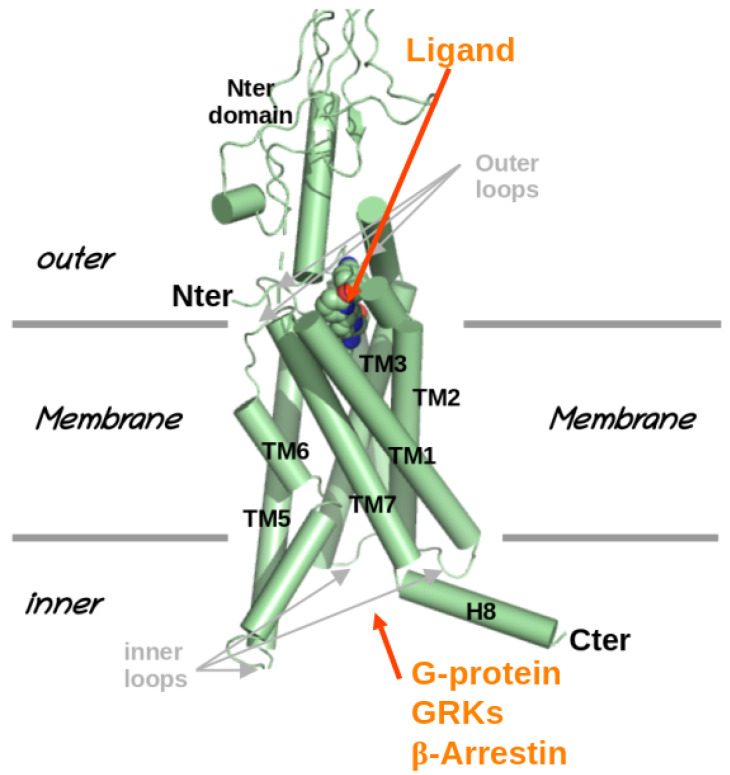
Structural organization of GPCRs. Example of the glucagon-like peptide-1 receptor (PDB: 7lcj).

Based on sequence homology and their functional similarities, the GPCR family has been divided into six classes [8] with three major classes (denoted as A, B, and C), where classes B and C are characterized, respectively, by large outer domains (100–500 amino acids) and very large outer domains (600 amino acids). Although GPCRs can recognize varied ligands and induce varied responses, their structural organization is remarkably similar. It consists of seven transmembrane (TM) helices linked by outer and inner loops, the outer loops (see Figure 1) involved in ligand recognition and the inner loops involved in the binding of one or more transducer proteins—G-proteins, GPCR kinases (GRKs), and β-arrestins. G-proteins are heterotrimeric proteins consisting of three subunits—α, β, and γ. Upon ligand binding, the GPCR is activated, which results in the dissociation of the α subunit of the G-protein αβγ heterotrimer, resulting in α and βγ. To date, 16, 5, and 12 different types of α, β, and γ subunits, respectively, have been identified [9], which leads to a large number of heterotrimer combinations, to relate to the vast number of consecutive physiological effects. Activated GPCRs can also interact with several GPCR kinases that phosphorylate several GPCR serine and threonine residues, triggering the binding of arrestin proteins, which block G-protein coupling and favor receptor internalization and modulate GPCR signaling in time [10,11,12].

With time, it has become more and more evident that GPCR signaling is much more complex than the simple model where the ligand activation of one GPCR triggers only one effect. First observations that different ligands could favor the activation of different pathways through the same receptor were reported in the late 1980s. Roth and co-workers [13] reported that several serotonergic receptors could be coupled to one or more transduction systems depending on their tissular localization, suggesting a receptor-associated bias. A few years later, Spengler et al. [14] observed that splice variants of the pituitary adenylyl cyclase-activating polypeptide (PACAP) receptor could alter response patterns of adenylyl cyclase and phospholipase C stimulation, suggesting a fine-tuning of signal transduction. Since then, there have been numerous reports supporting the occurrence of such modulation, referred to as biased signaling, and it is noteworthy that biased signaling does not seem exclusive of GPCRs [15]. GPCR-biased signaling [16] corresponds to the activation of different downstream pathways over others by different ligands of the same receptor. However, this can result from different mechanisms. The first one, referred to as the system bias, is that the receptor itself could be targeted in different tissues or subcellular localizations, associated with the differential expression of receptor subtypes or transducers. For instance, one such proposed mechanism is named the phosphorylation barcode hypothesis. It postulates that different states of phosphorylation of the receptor could shape the specific recruitment of inducers, thus biasing the signaling. The phosphorylation level may be controlled by ligand-specific receptor phosphorylation by GPCR kinases (GRKs) [17], depending on cellular localization [18], receptor expression, and ligands, as illustrated for the case of the chemokine receptor CXCR3, which are class A GPCRs [19]. According to IUPHAR [2], constitutive biased signaling mediated by splice variants [20,21,22] enters this category as well as the receptor bias associated with GPCR mutations [23], but is frequently referred to as receptor bias. A second one, referred to as ligand bias, corresponds to the differential signaling due to different ligands targeting the same receptor—same cell type, same cellular localization—but here again, some complexity may arise from the oligomerization state of the receptor [24]. GPCR oligomerization, and particularly hetero-oligomerization, has been reported as selectively inhibiting the G-protein pathway and activating the arrestin pathway. This has been observed for the kappa opioid receptor with NTS1R [25], CXCR7 with CXCR4 [26], OX1R, and CCK1R [27]. Note that, in the living organism, the observed functional selectivity is a combination of ligand and system bias. Finally, it has also been reported that, in some cases, the experimental setup itself could introduce an artificial bias or observational bias, for instance, focusing on a response at a time that is irrelevant to physiological processes (see [2]).

The way in which signal transduction is achieved through the membrane has been the subject of many studies [28]. The identification of the structure of the rhodopsin [29] and the ligand-activated β2-adrenergic (β2AR) receptor [30] in the 2000s has since been followed by the experimental resolution of GPCRs’ free agonist-bound or antagonist-bound structures. Similarly, the first structure of a GPCR bound to a G-protein obtained in the early 2010s has been followed by many others, thanks to the progress of cryo-EM. There are presently over 550 structures of GPCR complexes in the Protein Data Bank [31]. However, even if extremely valuable, all these structures only provide information on the most stable conformational states and do not provide a kinetic overview of the signal transduction. Techniques such as X-ray Free Electron Lasers (XFELs) [32], NMR in solution [33], Double Electron–Electron Resonance (DEER) spectroscopy, Bioluminescence Resonance Energy Transfer (BRET) [34], and Fluorescence Resonance Energy Transfer (FRET) [35,36,37] or molecular dynamics (MD) simulation techniques have been able to provide valuable insights in the structural mechanisms involved in signal transduction (see, for instance, [37,38]). Overall, the most remarkable ligand-induced structural change upon activation consists of a rotation and movement of the outer end of the sixth transmembrane helix, creating a pocket that can accommodate the binding of the transducers. This is facilitated by a displacement of helices 5 and 7 upon ligand binding. This mechanism seems general to all GPCR classes, with some specificities in terms of helix 6 movement/rotation (discussed in [39]). Several conserved sequence motifs are associated with the activation process, notably the CWxP and NPxxY motifs in helices 6 and 7, respectively [40]. Interestingly, these motifs mediate the activation pathway that extends from the ligand binding pocket to the cytoplasmic region responsible for G-protein interaction [41,42]. Some key ionic interactions have also been identified [43]. Moreover, water molecules have also been shown as possibly contributing to GPCR activation [44,45]. Several MD studies have investigated hydration waters within the TM region of GPCRs, have and shown the existence of a continuous water channel in the case of adenosine receptor type 2A [44,46]. MD simulations strongly suggest that water-mediated interactions are indispensable to achieve GPCR activation as, reported for the adenosine receptor type 2A [46] and the glucagon-like peptide-1 (GLP-1) receptor [47].

**Figure 2:**
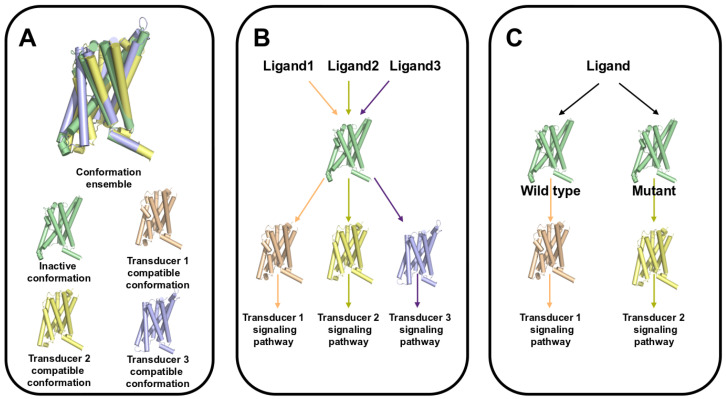
Conformational diversity of GPCRs and biased signaling. (**A**): Different conformations of GPCRs are represented, inactive (green) and active conformations (wheat), but also alternative conformations able to trigger different pathways by recruiting different transducers (yellow, indigo). (**B**): Ligand bias: different ligands of the same receptor are able to select different conformations recruiting different transducers, hence activating different pathways. (**C**): System bias: different pathways are activated upon the binding of the same ligand to different versions of the same receptor (mutants, isoforms, localization). Here, a mutation affects the selection of the conformation selection upon ligand binding.

However, accumulating evidence of biased signaling has imposed reconsidering the simple model of GPCR active and inactive states. Based on both experimental and in silico approaches, our understanding of the dynamics of GPCRs [39,48] is moving toward the model of a flexible system undergoing a complex conformational landscape encompassing not only the inactive and active states but also several intermediate states that are able to trigger varied signal transductions (Figure 2A). GPCRs can visit those intermediate states even without any ligand stimulation, but ligand binding or receptor modifications (mutations, isoforms, multimerization) can influence the probabilities of each state resulting in the biased signaling, as summarized in Figure 2B,C. The basal conditions favor inactive states, and intermediate and active states are visited with low frequencies, and upon agonist binding, the equilibrium is shifted toward the intermediate and active states. Ligand bias results from the selection of different active states resulting from the binding of different ligands on the same receptor, which in turn results in the recruitment of different transducers and then the activation of different pathways (Figure 2B). System bias results from modifications in the equilibrium of the states that can, for instance, lead a mutant receptor to select different conformations upon the same ligand binding, compared with the wild type (Figure 2C). Another example is that of a receptor constitutively adopting a preferred conformation, leading to the activation of a specific pathway, even without ligand binding.

The challenge of unraveling the complex signaling pathways triggered by GPCR activation has resulted in the search for means to stabilize each of the conformational states independently. Its feasibility has been demonstrated over 35 years ago in the case of the serotonin receptor [13], and has been well accepted since (see [49]). However, as discussed in [16,50], the development of such ligands is far from easy, being confronted with the need for means to measure the effect of a compound in a realistic-enough system, taking into account as many aspects as possible of the signaling, including locational, spatial, and kinetic aspects, as well as the possibility of off-target interactions, possibly including non-GPCR targets. With time, it has also been pointed out that full bias could be unreachable due to the flexibility of GPCRs [9]. Considerations outside GPCRs should also be taken into account. For instance, arrestin activation has been reported not only with but also without GPCR activation [51].

The understanding that a GPCR conformational landscape could be tuned depending on numerous factors has also raised interest in terms of drug development. In theory, biased compounds are expected to offer the perspective of developing functionally selective pharmaceutical drugs targeting specifically therapeutic signaling pathways while simultaneously avoiding adverse effects. Indeed, efforts have been reported for drugs targeting varied receptors (see [52]). In some cases, compounds able to target not only G-proteins versus β-arrestins but also G-protein subtypes have been reported [53,54,55]. However, if numerous compounds able to specifically bias GPCR response have entered the development process, only some have been successfully brought to the market [16,56], numerous adverse effects being observed in some cases, as for the development of drugs targeting the serotonin receptor [57].

As a result, efforts to push innovation in the field are increasing. This is true at the level of biophysical approaches to define new approaches to explore GPCR signaling, at the level of the wet lab to identify bench protocols suitable to probe compound selective effects, and also at the level of computational modeling, which now provides means to explore at the atomic level GPCR conformational changes undergoing the different signaling pathway activations and to assist the identification of biased compounds. Methods based on artificial intelligence (AI) are a great opportunity to better understand the structural mechanisms involved in biased signaling, since it is now a popular approach to model proteins at the atomic scale [58,59,60]. AI models have been successfully applied in a large number of tasks related to drug discovery, including target identification [61,62], hit identification [63,64], lead optimization [65,66], ADMET modeling [67,68], and protein design [69,70,71]. Here, we review recent advances obtained by in silico approaches to accurately model the varied conformational states of GPCRs, explore their dynamics, and assist biased ligand identification.

## 2. Computational Approaches to Explore the Structural Bases of Biased Signaling

### 2.1. Toward the Structural Modeling of Representative Conformations of GPCRs

Accurate modeling of the active, inactive, or intermediate conformations of a GPCR may prove valuable in deciphering the mechanisms of biased signaling since it can provide the basis for, first, a more comprehensive exploration of the biased signaling using MD simulations or, second, the search for compounds specifically targeting only one conformation. Recent progress in accurately predicting the structure of a protein from its sequence, notably since the release of AlphaFold2 (AF2) [72], RoseTTAFold [73], and ESMFold [74], and their latest evolutions capable of modeling large molecular complexes (like AlphaFold3 (AF3) [75], RoseTTAFold All-Atom [76], Chai-1 [77] and Boltz-1 [78]) open promising perspectives in that direction. AF3 is a significant update that incorporates modern deep learning techniques. The key improvement is a new structure prediction module based on diffusion that can predict the position of individual atoms, instead of residue frames in AF2. It makes it possible to predict various types of structures, with the ability to model molecular assemblies containing modified proteins (for instance, with post-translational modifications), DNA, RNA, small molecules, and ions. AF3 was shown to predict GPCR structures more accurately than AF2, but it struggles to predict the structure of ligand–GPCR complexes [79]. Still, AF3 was useful to model the T1 isoform of the receptor tyrosine kinase in the interaction with various activating ligands, subsequently used to design a selective antagonist for this specific isoform [80]. However, these approaches have not been designed to truly capture the dynamic properties of proteins and do not generate models that address the diversity of relevant conformations. The quality of AF2 predictions was even found to decrease for proteins with more conformational diversity [81]. With respect to the specific aspects of GPCR signaling, model prediction should meet the requirement of being able to distinguish between relevant subtle conformational changes.

**Figure 3:**
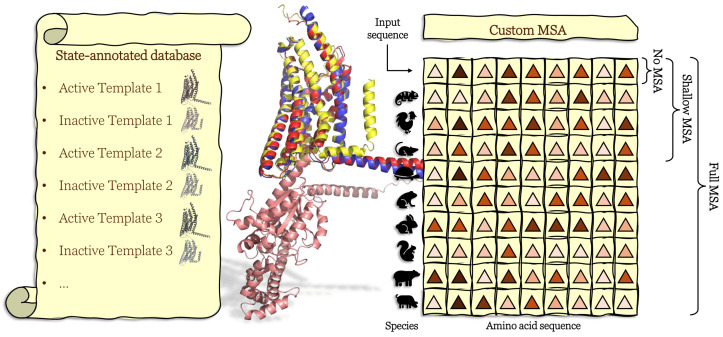
Illustration of modified protocols to generate diverse GPCR conformations with AlphaFold. Templates are retrieved from a state-annotated database, replacing the default database. Depending on the protocol, different depths of MSA can be used. The different colored triangles in the MSA table represent different amino acids. Modeling the structure of the GPCR-Gα subunit complex with AlphaFold-multimer can lead to a higher-quality GPCR structure. In this example, the Succinate receptor 1 structures are retrieved from GPCRdb and GproteinDb, including the active and inactive models in blue and yellow, respectively. The GPCR structure modeled with a Gαs subunit using AlphaFold-multimer is represented in red (Gαs subunit in salmon).

To address this issue, protocols have recently been developed to guide the structure generation process toward the desired GPCR conformation, enabling researchers to rationalize ligand-induced biases. Recent efforts have focused on the particular use of multiple sequence alignment (MSA) and templates with AlphaFold, as described in Figure 3.

Heo et al. [82] have proposed a protocol to guide AF2 to model both active and inactive structures of a GPCR. The input consists not only of the sequence of the GPCR of interest but also of the template of an active or inactive structure together. No multiple sequence alignments (MSAs) are provided. Their protocol can take advantage of an annotated database of active and inactive GPCR templates (224 and 309, respectively) extracted from GPCRdb [1], keeping one representative GPCR per cluster at a 100% sequence identity threshold. Other protocols have proposed replacing the default AF2 alignment sequence with a shallow MSA, arguing that it enables the sampling of diverse conformations [83,84]. Notably, Sala et al. have shown that it is better to use a shallow MSA than no MSA to accurately model loops in GPCRs [83] since incorporating genetic information may help to overcome the issue of lack of conserved residues. Lee et al. [85] have recently compared different protocols to model GPCRs, including the protocol by Heo et al. [82] and others based on AlphaFold-Multimer [86], which involve modeling the GPCR in the interaction with the entire G-protein or only its G-α subunit. They found that, for class B1 GPCRs, modeling the GPCR in the interaction with the α chain significantly increases the accuracy for the extracellular domain. This is particularly interesting because it suggests that AlphaFold-Multimer can capture information from the binding partner of the GPCR to refine its conformation. Therefore, it could be worthwhile to explore this idea to extract structural insights related to bias signaling. For instance, GPCR structures could be compared when it is modeled with a G-protein or a β-arrestin partner. Now that it is possible to predict the structure of large assemblies containing small molecules and proteins [75,76,77,78], a similar approach could be followed, modeling differently biased ligands with GPCRs to better understand the biased signaling mechanism. As an outcome of this progress, databases containing experimental structures and models generated following the protocols introduced above have been built. GPCRdb has already included predicted structures (active and inactive) to overcome the structural data scarcity issue, and it also comprises annotation related to functional activity [1,87]. In addition, GproteinDb contains G-protein–GPCR complexes modeled with AlphaFold-Multimer, including 5595 out of 6800 theoretical human Gα-protein–GPCR complexes [88], after matching 425 GPCRs with 16 different Gα-proteins. In a structural study of biased signaling, Seyedabadi et al. [89] reported that general conformational differences between GPCRs bound to either G-proteins, β-arrestin, or GRKs could not be identified after a detailed analysis based on experimental structures. This highlights the opportunity to model the interactions of GPCRs with their partners to better understand the structural implications of biased signaling. However, one must remain cautious and properly assess the quality of the structures modeled with these tools. GproteinDb contains structures with AlphaFold quality metrics above given thresholds [88], but this does not guarantee that the models are truly accurate. For instance, Terwilliger et al. reported that 10% of residues predicted with high confidence by AlphaFold show an error greater than 2Å [90]. In the future, it may be expected that modeling GPCR structures in greater detail will become possible, thanks to template databases annotated with more than two states. It remains that recent progress in structure modeling is rapidly extending the structural data available to all GPCRs, with the perspective to have in the near future a full collection of inactive and active conformations possibly enriched by intermediate ones.

### 2.2. Molecular Simulations to Unravel Biased Signaling

Molecular dynamics (MD) simulations are now recognized as a powerful technique for capturing protein structural motions at the atomic level and can complement conventional experimental techniques for which sampling new conformational states might prove difficult. MD approaches play a tremendous role in gaining insight into the interactions that govern the conformational states. These approaches also seek to establish a correlation between the stability of particular conformers and the effect exerted by specific pharmacological ligands. MD simulations have been extensively applied to study a wide range of biological systems, including G-protein coupled receptors (GPCRs). Moreover, several MD studies provided valuable insights into the dynamical changes of GPCR class A when binding to biased agonists [91,92,93,94,95,96,97]. On the other side, computational studies on biased signaling of other GPCR classes and biased mutations have been limited and still need to be explored [98,99,100].

#### 2.2.1. Contribution of Transmembrane Helices in Biased Signaling

**Figure 4:**
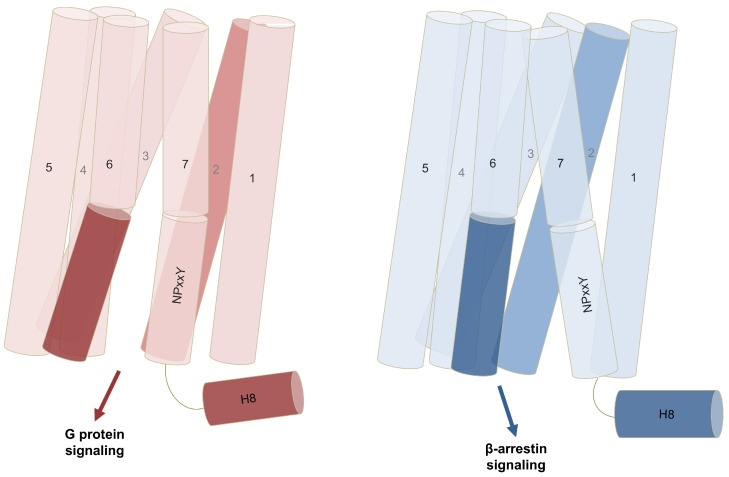
Conformational changes of TMs upon G-protein or β-arrestin signaling. G-protein signaling in most of the GPCRs showed an outward movement of TM6. On the other hand, β-arrestin signaling was reported to be accompanied by structural alterations in TM7 and H8, reducing the distance between TM7 and TM2.

Many studies have shown that GPCR signaling is mediated by the seven transmembrane helices. Important conformational changes of TMs upon biased signaling have been observed among in GPCR class A. It has been shown that either β-arrestin signaling or G-protein signaling is conducted through displacements and structural features of TMs. A common feature is the outward movement of TM6, which favors G-protein binding. Moreover, an inward shift of TM7, holding the NPxxY motif, toward TM2 at the intracellular end, reducing the distance between TM2 and TM7, is known to induce β-arrestin binding. Additionally, this shift of TM7 is accompanied by a conformational change in helix 8 (H8) and ICL1. These structural changes play a vital role in biased signaling in β2AR, as previously reported [101,102,103,104] Figure 4.

One way to unravel biased GPCR signaling is to use ligands that encourage receptor interaction with one type of signal transducer, either G-proteins or arrestins. Biased ligands were first identified as being involved in the activation of various G-protein families (such as G$\alpha_{s}$, G$\alpha_{i}$, G$\alpha_{q}$, and G$\alpha_{12/13}$) and even specific Gα subtypes within the same family; biased ligands are now recognized for their capacity to generate unique signaling profiles [105,106]. By selectively engaging either G-proteins or β-arrestins, these ligands are also described as functionally selective ligands, highlighting their importance in the modulation of receptor signaling. Several computational studies have investigated multiple GPCR signaling pathways using various biased ligands. The important findings presented in these studies are discussed below.

The signaling of multiple G-proteins was reported in a number of structural studies in order to identify the key elements involved in biased signaling and the conformational changes that result consequently [107,108,109]. In a recent study, MD simulations of relaxin family peptide receptor 3 (RXFP3) bound to biased (14s18 and d(1-7)14s18) and unbiased (relaxin-3 and peptide 4) ligands were applied to elucidate the molecular mechanisms that govern G-protein and β-arrestin signaling [108]. Two different conformations (open and closed) of the receptor were obtained for unbiased and biased ligands, respectively. The open conformation induced by unbiased ligands, relaxin-3 and peptide 4, was adopted in response to the movements of TM6 and TM7 and the interactions formed with the loop connecting them, which resulted in the recruitment of β-arrestin. However, biased ligands involved different binding modes with TM2, TM5, and TM6 that facilitated G-protein binding. Kling et al. [109] carried out MD simulations for β2AR complexed with G$\alpha_{s}$ and BI167107 and the dopamine D2 receptor (D2R) complexed with G$\alpha_{i}$ and dopamine. They found that, in the case of the β2AR complex, residues in TM3, TM6, and TM7-H8 are preferentially bound to G$\alpha_{s}$. For the D2R complex, the formation of salt bridges between TM4 and TM5 and the α4 and α5 helices of G-protein, respectively, led to the strong binding with G$\alpha_{i}$ [109].

MD simulations of the vasopressin V2 receptor (V2R) with the MCF14 ligand revealed distinct conformational changes in the TM7 NPxxY motif and H8 as a result of Gs-biased signaling [110]. These conformational changes were detected by calculating the interhelical distances TM2-TM7, TM1-TM7, and TM1-H8. The authors observed that I130N mutation caused a structural alteration at the TM7 NPxxY motif and H8 close to the one obtained by MCF14 due to the formation of additional H-bonds between TM3 and NPxxY motifs. The authors proposed that these conformational changes are transmitted allosterically through the Na^+^ binding site in TM2. Plazinski et al. [111] performed coarse-grained MD simulations and demonstrated that the stereochemistry of ligands can impact the signaling pathway, as shown for the isomers of 4’-methoxy-1-naphtylfenoterol (MNFen), (R-R) and (R-S) bound to (β2AR). The S-configuration induced an outward movement of TM6 that resembles another conformer bound to β-arrestin (PDB: 3SN6). In another MD study of β2AR bound to formoterol, it was shown that the signal is conducted to the β-arrestin interface through an allosteric pathway involving residues in TM5, TM6, and TM7 [101].

Energy calculations of D2R [112] showed the importance of TM3 in G-protein bias upon binding of the G-protein biased agonist MLS1547, while in the case of binding of the β-arrestin biased agonist UNC9975, TM1 and TM6 were critical for β-arrestin binding. The authors used the Molecular Mechanism–General Born Surface Area (MM–GBSA) method [113], which indicated that UNC9975 bound more strongly to D2R than MLS1547. Moreover, they showed from the secondary structural analysis that the formation of kinks in the transmembrane domains influences the recruitment of either G-protein or β-arrestin. The formation of kinks in TM2, TM4, and TM7 led to recruiting G-protein, while β-arrestin binding was induced by a kink in TM5 in D2R. Another recent MD study investigated the molecular mechanism of the biased G-protein agonist SK609 and the unbiased agonist PRX in the dopamine D3 receptor [114]. Principal component analysis (PCA) of MD trajectories captured the conformational changes occurring in the TM region. A large movement and a more pronounced tilt in TM3 were observed in the case of PRX compared with SK609, increasing the flexibility of TM3 in unbiased signaling. G-protein biased signaling was induced by hydrophobic interactions between SK609 and TM5 and ECL2 [114]. Interestingly, this was previously detected with MLS1547, another G-protein biased D2R agonist, which formed hydrophobic interactions with V190 and F189 of TM5 [115].

Nivedha et al. [116] carried out MD simulations for the Angiotensin II type 1 receptor (AT1R) bound to various agonists. They performed an inter-residue distance analysis that showed the importance of microswitches that control the activation and inactivation of the receptor. The study showed that β-arrestin signaling involved key contacts mainly situated in TM7 and a decrease in the distance between TM3 and TM6. Additionally, AT1R studies discussed the role of TM7 conformation for β-arrestin binding, which is closely related to the presence of Y292 in the binding pocket of AT1R [117,118]. Another AT1R study explained that the absence of a chemical group in the biased ligand could play a pivotal role in biasing the signal. They evaluated this by the absence of N-terminal arginine in G*_q_* biased agonists, which resulted in the lack of any interaction between the ligands and the extracellular part, leaving a wide area for the movement of the C-terminal phenylalanine to adopt a stable conformation [119].

Other studies demonstrated that the dimer form of the GPCR can influence the signaling upon ligand binding. Chen et al. studied the effect of the DAMGO agonist on a GPCR heterodimer (μOR/δOR heterodimer) combining Gaussian accelerated molecular dynamics (GaMD) simulation and a protein structure network (PSN). The agonist exhibited high affinity to β-arrestin with signal transduction through TM6 and TM7 [102]. Furthermore, in another study of the chemokine receptors (CXCR4) dimers, it was demonstrated that the dimers adopted an open conformation that recruited β-arrestin, while the homodimers were found to bind strongly to G*_o_* protein [120]. These results were further confirmed by the open models of a CXCR4 dimer that were generated using AlphaFold-multimer [120].

MD simulations were recently used in combination with deep learning techniques to identify the signaling pathway by which GPCR transmits the signal. Chen et al. [121] carried out accelerated molecular dynamics simulations followed by ligand binding mode analysis, potential of mean force (PMF) analysis, and an interpretable convolutional neural network-based classification model to explore biased signaling in β2AR. In this study, G-protein biased signaling induced by TRV130 was associated by a large outward movement of TM6, while the recruitment of β-arrestin by c involved inward conformational changes of TM7 and H8, which led to an inactive-like conformation of the receptor. This classification model enabled the identification of the key residues that are responsible for biased signaling. These residues were particularly found in the intracellular ends of TM5, TM6, and TM7 [121]. Moreover, TRV130 was found to induce conformational changes in TM6 that were caused by binding to TM2 and TM3 in the μ-opioid receptor [43]. Similar structural alterations of TM6 were also reported for the PZM21 agonist when bound to the μ-opioid receptor [122,123].

From these MD results, some general structural trends start to emerge. These findings suggest that G-protein signaling can be associated with closed conformations of the receptor, while β-arrestin signaling rather requires open conformations. These conformations are adopted as a result of the movements of TM helices, especially TM6 and TM7. An outward movement of TM6 and an inward movement of TM7 are commonly featured in class A GPCRs. Their movements consequently affect their distances to their facing TMs, such as TM2 and TM3. The movement of TM7 is recurrently coupled with the displacement of H8, leading to β-arrestin signaling [108,110,121]. Additionally, certain biased ligands can also induce the formation of kinks in TMs in response to the recruitment of a specific transducer [112]. Moreover, combining MD simulations with several techniques, such as free energy or machine learning, gives insights into the mechanism of biased signaling and allows the identification of the main contributors to the signal transmission that were not previously recognized by traditional methods [121,124].

#### 2.2.2. Role of Loops in Biased Signaling

GPCRs have a conserved structure of seven transmembrane domains that are linked by six loops, three extracellular (ECLs) and three intracellular (ICLs) [125,126]. Several MD studies on GPCRs investigated the impact of the structural rearrangement of loops in signal bias [95,103,104,124,127,128]. It was reported that ergotamine, which recruited β-arrestin in the 5-HT2B serotonin receptor, induced the closure of ECL2 [129]. Additionally, ECL2 had a pivotal role in β-arrestin signaling in β2AR as it has been shown that the ECL2 residue L209 is essential for the recognition of biased active states by the LDS agonist [103].

It was discovered that β2AR’s intracellular loop 3 (ICL3) is crucial for interacting with G*α_s_* and altering the conformation of the α5 helix of G*α_s_*. The formation of the inactive state of the GPCR complex is controlled by the induced conformational change of the α5 helix [104]. Other computational studies proved the presence of a dynamic equilibrium between ICL3’s closed states, which block the G-protein binding site and render the receptor inactive, and its open states, which permit interactions between receptors and effectors and promote receptor activation. In particular, it was demonstrated that ICL3 in β2AR is biased to open states by a native peptide derived from the C terminus of the G*α_q_* subunit, thereby resulting in receptor activation [128].

Free energy calculations were carried out by Mafi et al. [124] using umbrella sampling of human μOR to investigate the effects of biased signaling upon binding of the TRV130 bias G-protein ligand and unbiased morphine agonist. The study revealed the importance of ICL2 and ICL3 in recruiting $G_{i}$ upon TRV130 binding. The study showed that charge–charge interactions are crucial for $G_{i}$ signaling. Salt bridge interactions were formed between residues of ICL2 and ICL3 and the G$\alpha_{i}$ subunit. Additionally, the study demonstrated that the binding of morphine to phosphorylated μOR led to the fixation of β-arrestin by forming strong hydrogen bond interactions with ICL2 and ICL3 residues. Further, the study showed that the obtained conformation was energetically stable. It thus denoted the critical role of ICL2 and ICL3 in coupling β-arrestin with polar contacts. Surprisingly, the recruitment of G$\alpha_{i}$ protein in μOR by TRV130 involved the same residues of ICL2 and ICL3 that formed strong interactions while binding to morphine and led to the recruitment of β-arrestin in the case of phosphorylated μOR. These observations suggest the presence of a competition between G*_i_* protein and β-arrestin on the same binding site, even though their binding results in contrasting effects [124].

These findings offer new understandings of the dynamics of GPCR bias signaling by demonstrating a link between the structural rearrangement of the loops and the recruitment of G-protein or β-arrestin. The contribution of ICLs in biased signaling appears to be more prominent than ECLs. ICLs strongly bind to G-protein or β-arrestin by forming electrostatic or polar interactions. Additionally, the phosphorylation of the receptor plays a significant role in binding G-protein or β-arrestin through ICL interactions. Furthermore, the opening or closure of loops can act as a moderator for biased signaling as a result of the conformational changes occurring in the transmembrane helices [130].

#### 2.2.3. Impact of Mutations on Biased Signaling

Naturally occurring mutations can arise from genetic modifications or changes in DNA sequences, leading to structural and functional consequences in the receptors. These mutations are among the well-known causes of numerous human diseases [131]. These mutations can be inherited from parents or caused by environmental factors like chemicals or radiation, and they can also take place spontaneously during DNA replication [132,133,134]. The effects of mutations on receptor functionality can vary significantly. They can modify the interactions between the receptor and its ligand, affect its activity levels, or disrupt signal transduction pathways. In GPCRs, mutations can disrupt the signaling process and render the receptor inactive or modify the signaling pathway, thereby altering the activation outcome [23,131,135].

Computational techniques play a crucial role in examining mutations across various receptor types associated with diverse diseases. Notable progress has been made through molecular modeling of GPCR mutations for either characterizing the mutations, investigating the signaling pathway, or examining the activation mechanism [136,137,138]. A study combining MD simulations with in vivo and in vitro data was performed to explore the mechanism of a novel mutant I423T targeting the follicle-stimulating hormone receptor (FSHR) and leading to primary ovarian failure. This mutation was found in TM2 and selectively activated β-arrestin, and this effect is strongly related to the interactions formed between TM1 and TM6 [139]. The I423T mutation specifically impacted receptor activation. Notably, the mutation at residue 423 is situated at the interface of TM2 and the first extracellular loop, where a group of five amino acids (residues 422 to 426) forms a turn at the extracellular region of TM2. The conformational stability of this turn is crucial for the precise regulation of hormone binding and receptor activation [140].

Furthermore, lab-generated mutations are often produced in GPCRs to investigate the importance of specific residues in signaling and compromising the structure and function of the receptor. GPCR biased signaling not only results from ligand binding but also can result from these mutations, influencing some key residues [23,141,142,143,144,145]. Although the molecular mechanism of biased signaling remains elusive, there has been increasing proof from previous studies that biased ligands and mutations trigger particular downstream signaling pathways by maintaining specific receptor conformations [16,106,146,147]. These studies either focused on a localized illustration of the biased conformations or offered a static description of these receptors [148,149,150,151]. Therefore, the use of MD simulations gives a complementary edge for investigating a range of functional conformations induced by certain mutations that contribute to receptor signaling [96,97,152,153].

In a recent study using GaMD simulations to distinguish the effects of a single mutant (Y219^5.58^A) and triple mutants (T68^2.39^F, Y132^3.51^G, and Y219^5.58^A) in β2AR systems [154], the authors applied an unsupervised machine learning algorithm, the Gaussian mixture model (GMM), that clusters MD trajectories in order to analyze the extracellular and intracellular differences in transmembrane helices resulting from the single and triple mutations. It was found that a single mutation Y219^5.58^A diminishes the binding affinity for G-protein coupled receptor kinases (GRKs), thereby reducing the receptor’s interaction with β-arrestins and resulting in a bias toward G-protein signaling. Conversely, the triple mutations (T68^2.39^F, Y132^3.51^G, and Y219^5.58^A) impede G-protein signaling, leading to a preference for β-arrestin engagement. The receptor adopted an open conformation in the single mutant, which enabled G-protein binding. On the other hand, the triple mutant favored a closed conformation that hindered G-protein binding [144,154].

In another MD study, Sanchez-Soto et al. [152] assessed the effects of specific single-point mutations, I184^ECL2^A, F189^5.38^A, and V190^5.39^A, on a D2R signaling activity in the presence of a G-protein biased ligand, MLS1547. These mutations completely blocked the G-protein signaling pathway, which led to the loss of MLS1547 biased activity. These findings suggest the presence of structural fingerprints in a D2R pocket that are responsible for ligand efficacy and signaling bias. In contrast, when the signaling responses of the mutant D2R were assessed with the unbiased agonist dopamine, the I184^ECL2^A and V190^5.39^A mutants exhibited diminished binding affinity for dopamine. The mutants were also less potent in recruiting both G-protein and β-arrestin; however, they did not lose functional efficacy in both recruitments. Conversely, the F189^5.38^A mutant demonstrated a decrease in dopamine binding affinity and potency for G-protein-mediated signaling, similar to the other two D2R mutants; however, it completely lost efficacy in stimulating β-arrestin recruitment. Notably, the capacity of dopamine to maximally activate G-protein-mediated signaling remained intact [152].

These findings demonstrate the influence of mutations on GPCR dynamics and signaling, as well as their function. Biased ligands, as previously discussed, may represent good drug candidates due to their favorable effects of modulating signaling pathways in wild-type GPCRs. Even with the advances in computational techniques nowadays, there is still a need to design more ligands for biased mutant GPCRs. Additionally, the exploration of the signaling and structural characteristics of biased mutant GPCRs will enhance our understanding of the structure–function relationships of these receptors, thereby facilitating the development of therapeutics targeting specific mutant-related sites to treat various diseases.

## 3. Predicting Ligand Bias

Computational methods have been widely applied in studies related to GPCRs to perform virtual screening of small-molecule libraries with molecular docking [155,156,157,158,159,160,161] or to predict the affinity between a small molecule and its GPCR target with expensive free energy calculations [162,163,164]. However, the most accurate physics-based methods are often resource intensive, hindering their use at large scales, and consequently, machine learning has become more popular, thanks to its efficiency and its ability to learn from large amounts of data [165,166]. In contrast, only a smaller number of studies have attempted to go further to predict the biased signal induced by the ligand binding. However, it is crucial to predict the signaling pathways triggered by the ligand binding instead of only predicting the affinity. The studies introduced in this section have leveraged machine learning classifiers to tackle this problem with varying degrees of detail in ligand-based and structure-based frameworks.

### 3.1. Overview of Established Methods

A first aspect is the classification of ligands as agonists or antagonists. Random forest (RF) has recently been used for this aim [167,168]. Oh and co-workers [167] considered ligand information only, using two consecutive models in turn, the first predicting whether the ligand binds to GPCRs and the second whether the ligand is agonist or antagonist, without restriction to a single GPCR subfamily. The required annotated data were retrieved from GtoPdb [169] and the CODA network database [170]. To encode the 4590 ligands from these databases, they selected 990 molecular descriptors generated by Dragon [171] as inputs of their RF classifiers. Their combined model achieved a ROC AUC of 0.795 after a leave-one-out cross-validation procedure. Goßen et al. also used RF models (they developed both ligand-based and structure-based classifiers) to predict antagonists of the human adenosine receptor type 2A [168]. Their structure-based model takes a protein–ligand interaction fingerprint as input, extracted from a pose generated by molecular docking with Glide [172]. They validated their predictions in vitro, leading to the identification of an antagonist (affinity of 310 ± 23.4 nM), dissimilar to the ChEMBL database (maximum Tanimoto similarity of 0.33). Similarly, Jiménez-Rosés et al. performed molecular docking with Autodock Vina [173], followed by scoring with RF and XGBoost (XGB) models to identify the interactions that help discriminate between agonist and antagonist [174]. DeepREAL is a deep learning framework that performs three-way classification (agonist, antagonist, nonbinder) [175]. It provides the advantage of considering both the GPCR and the small molecule, so it does not need to be retrained when studying a new target. The authors leveraged a multi-step pre-training strategy to tackle the data scarcity issue, including pre-training the protein sequence embedding and the drug–target interaction modules. Then, they fine-tuned their model on the three-way classification task. Notably, the model was validated when used in a challenging scenario (tested with small molecules dissimilar to the training set, with opioids), outperforming other models with a ROC AUC of 0.71. However, these approaches only aim to differentiate between agonist and antagonist, not directly predicting the pathway activated by the ligand binding, which reduces the applicability of such methods in real-world projects.

Beyond the oversimplified agonist/antagonist classification, other studies have built machine learning models to predict G-protein and β-arrestin bias. BiasNet was trained to predict G-protein or β-arrestin bias in a ligand-based framework, independently of the GPCR target [176]. A total of 727 bias cases (including 547 unique ligands and 61 receptors) from BiasDB [177] were used to train five models: RF, XGB, Support Vector Machine (SVM), Multilayer Perception (MLP), and Directed Message Passing Neural Network (D-MPNN). The models were combined with 15 different types of molecular descriptors, including various fingerprints and physicochemical features. They identified secondary amines and aromatic amines as the most important fragments that contribute to the prediction, suggesting that these fragments contribute more to the β-arrestin bias than the G-protein bias. However, this observation must be subject to caution given the small size of their dataset and the simplicity of their target-agnostic approach. Kumar et al. [178] went further in subtlety to predict ligand bias divided into four classes: $G_{i/o}$, $G_{q/11}$, $G_{s}$, and β-arrestin bias. Their study focuses on the advantage of combining multilevel features, taking advantage of non-covalent interactions’ derived features, binding pocket geometry information, and bioactive conformation of the biased ligand. Their model achieves a ROC AUC of 0.844 on an external test set. Additionally, they identified features that might be involved in the different biased signaling pathways. In particular, they found that $G_{i/o}$ and $G_{s}$ biases are mainly related to two different interaction features, both localized on the same TM6 domain, therefore underlying the subtlety of interaction network distinction between differently biased ligands. They applied their model to the Mas-related G-protein coupled receptor member X2 (MRGPX2) and discovered an antagonist ($IC_{50}$ of 15.6 μM). Therefore, this model can be used to identify key interactions and achieves reasonable performance, which is promising for more applications in the future.

### 3.2. Emerging Trends for Ligand Bias Prediction

**Figure 5:**
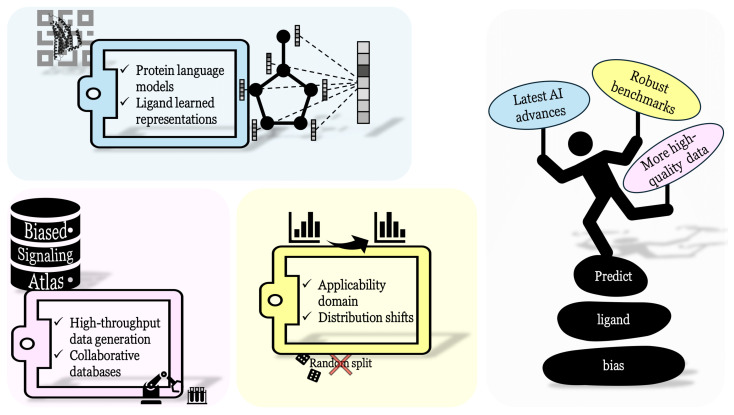
Depiction of the challenges related to ligand bias prediction.

To be useful for real-life applications, the predictive models must be able to capture the underlying complexity of ligand bias (i.e., discriminate between many signaling pathways), and their applicability domain must be expanded. This requires (1) more high-quality data for reliable predictions in a larger chemical space, (2) robust benchmarks to compare the performances in a rigorous way, and (3) incorporating the latest advances in AI to make the most of available data, as represented in Figure 5. These challenges are discussed below.

To address the first issue, data collection initiatives are ongoing. The Biased Signaling Atlas [179] gathers biased ligand data. Currently, it includes 8956 ligand-receptor-pathway activities and will keep growing in the future with the contribution of more researchers. In this respect, guidelines were defined to ensure the consistency of data produced by various laboratories worldwide [2]. Presently, training datasets are assembled from diverse databases [169,170,177,180] or directly from the literature [181], thus requiring a tedious procedure. High-throughput methods would be particularly valuable to generate data related to ligand bias given the modest amount available. We anticipate that such methods will become an area of growing interest, as exemplified by the recent large-scale identification of biased ligands for the δ, μ, and κ-opioid GPCRs with DNA-encoded libraries (DELs) containing 9216 ligands [182].

Robust benchmarks must be established in the field to reliably assess and compare the performances of the models used to predict ligand bias. Apart from DeepREAL [175] and GPCR-IPL [178], the studies introduced above do not consider the applicability domain in depth to evaluate their models (i.e., identifying the chemical space where the model is confident), making them unsuitable for use in a real case [183]. Moreover, such models are not properly evaluated under more challenging (and realistic) scenarios involving train–test distribution shifts [184] (i.e., assessing the model performance with ligands dissimilar to the training dataset). For these predictive models to have a positive impact, robust benchmarks that take into account these issues must be adopted.

Finally, recent advances in AI, already successful in other fields of biology, should be incorporated into ligand biased prediction models to improve predictions. When not limited to ligand-based models, some models have tried to extract valuable information from the GPCR target. However, there is a trade-off between sequence-based GPCR encoding [175] that can learn from widely available data and structure-based GPCR encoding [178] (or even encoding of the GPCR–ligand complex), which suffers from scarce data but may contain richer information. With these considerations in mind, protein language models (PLMs) trained on billions of proteins [74,185,186] are a key opportunity since they are able to encode the GPCR sequence, capturing rich evolutionary information. This encoding could replace less efficient others, while not requiring more structural data that are costly and time-consuming to produce. Similarly for the ligand encoding, learned representation [187] (directly learning how to efficiently encode the ligand, as opposed to traditional molecular descriptors or fingerprints) like graph transformers [188] or graph neural networks (GNNs) may better encode the chemical information relevant to predict ligand bias. The latter was used in some of the studies presented before [175,176] and has already led to promising results for the GPCR affinity prediction task [189]. These AI trends will be beneficial to ligand bias prediction, and could even facilitate biased ligand design.

## 4. Prospective Methods to Discover Biased Ligands

Recent studies integrating experimental data and in silico modeling have shown that a rational design of biased ligands is now possible. The integration of AI-powered approaches can make the design process faster and more efficient, provided that the right methods are used and that we learn from successful traditional methods. To this end, we propose to analyze rational studies of a biased ligand design to suggest the best ways of using state-of-the-art AI tools.

McCorvy et al. combined experimental (biochemical assays including wild-type and mutant proteins) and in silico (homology modeling, molecular docking, molecular dynamics) experiments to understand the structural rational behind biased ligands for the D2R [190]. A key feature of their study is that they generalize their findings to the other GPCRs of the aminergic family, therefore making it possible to apply their design strategy to more GPCRs. They found that the interaction of the ligand with ECL2 favors the β-arrestin bias while the interaction with TM5 contributes to the G-protein activation. To go further in the understanding of the biased signaling, they identified specific residues in these domains that allow D2R-ligand interactions that contribute the most to the bias. Based on these considerations, they designed β-arrestin biased ligands, including one compound achieving a remarkable bias factor of 20 (with quinpirole considered as the reference).

However, its design was guided by chemical intuition, speculating that the β-arrestin bias enhancement of a D2R partial agonist could be obtained from the methylation of its indole ring because it would promote the interaction with hydrophobic residues in ECL2. Alternatively, data-driven approaches appear to be a good solution to be used as an alternative to an expert-guided design. Generative AIs are particularly well suited to this kind of problem, having already been widely successful in other fields such as text and image generation [191,192,193,194]. Diffusion models have become increasingly popular for a small molecule design [195] and represent a promising opportunity for a biased ligand design. In short, diffusion models learn to reverse the noising process, thus becoming able to generate new molecules from sampled noise. There are already a lot of these models that allow for designing small molecules in a structure-based framework, integrating protein target conditioning [196,197,198,199,200]. However, these generative models could be adapted for a structure-based design of biased ligands if they can be guided to target specific hotspots, i.e., residues provided by the user. In that respect, Wu et al. developed a diffusion model that integrates this feature via an interaction prompt, which specifies the hotspot residues and the interaction type [201]. We anticipate that the relevant incorporation of these tools, combined with well-established methods, will be valuable for a structure-based biased ligand design.

**Figure 6:**
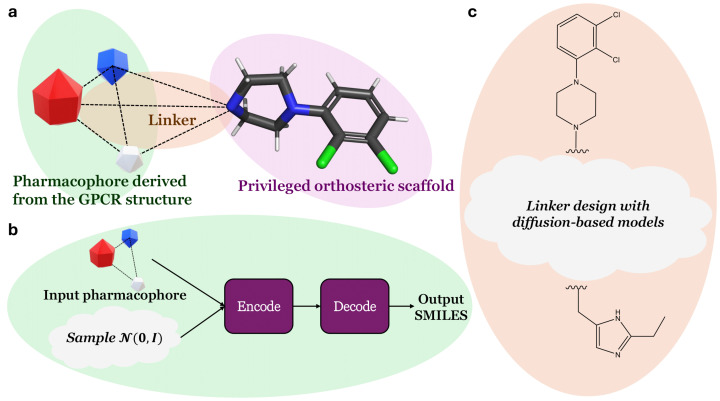
(**a**) Design strategy incorporating AI methods to identify biased ligands for the D2 dopamine receptor, inspired by the structure-based approach by [202]. (**b**) Schematic representation of the workflow to generate a scaffold to interact with an allosteric site of D2R, leveraging the encoder–decoder architecture conditioned with a pharmacophore developed by [203]. The pharmacophore can be derived from the structure of the allosteric pocket. (**c**) Generative diffusion model to design a linker. A linker can be generated with a diffusion-based model [204] to link two scaffolds designed to interact with specific D2R residues involved in biased signaling.

Regarding the D2R case, Männel et al. reported an alternative method to discover biased ligands, considering the orthosteric site and an allosteric pocket [202]. Based on molecular docking results, they noticed that known biased ligands occupy both sites. Therefore, they virtually screened a library of 13,000 compounds with a 2,3-dichlorophenylpiperazine moiety (known to interact with the orthosteric site) and only retained the best candidates with a pose involving additional interactions with the allosteric site. This strategy led to the discovery of biased ligands, including one that only activates the β-arrestin pathway. Again, generative AIs could prove useful in this scenario, if the conditioning of the model is well chosen. For instance, Zhu et al. constrained the generative process of their transformer-based framework with a pharmacophore, which can be derived from the structure of the target and known binders [203]. This approach is particularly well suited in this case since it makes it possible to generate small molecules that simultaneously satisfy more than one condition (interaction with both allosteric and orthosteric sites). The AI-based strategy discussed here to design biased ligands for D2R is represented in Figure 6.

The structure-based approach has also proved successful in the case of biased peptide design, as demonstrated by a recent study [205] that led to the discovery of G-protein biased peptide agonists of the apelin receptor (APLNR). They leveraged peptide–APLNR–$G_{i1}$ cryo-EM complex structures and functional experiments to understand the detailed structural mechanism of biased signaling. They identified two hotspots that contribute greatly to the biased signaling, and they highlighted the role of the peptide interaction with TM6 and TM7 toward G-protein bias. These structural insights could have been exploited to design biased peptides with generative AI. Inspired by the success of protein design frameworks [206,207,208], generative models have been developed specifically to design peptides [209,210,211]. Once released, generative models like PepHAR [212] will be of great value because it allows the user to specify hotspots before designing missing residues, therefore incorporating structural knowledge into the design process.

Linker design approaches based on diffusion models can also be applied in biased ligand design. For instance, DiffLinker can connect different fragments to design valid small molecules, and can also be conditioned with the target pocket [204]. This linker approach is particularly well suited to bivalent biased ligand design. Lensing et al. successfully designed bivalent biased ligands that target the human melancorotin-4 receptor (hMC4R) dimer, linking an agonist and an antagonist together to selectively activate the G-protein pathway [213,214].

Given the complexity of structural insights into ligand bias, phenotypic approaches may appear to be a practical alternative. The successful design of dual agonists of the human glucagon receptor (GCGR) and the glucagon-like peptide-1 receptor (GLP-1R) was recently reported [215], and the method employed in the study can be slightly adapted to design biased agonists. The directed evolution workflow was guided by an oracle trained to predict the affinity of candidate peptides against both receptors. Instead, training the oracle with data directly related to G-protein and β-arrestin pathway activation (i.e., simply changing the targets of the multitask framework) would guide the design process toward biased agonists. To go further with the latest diffusion-based models, ProteinGenerator [216] can be combined with such an oracle to guide the diffusion process, and adapting their multistate design strategy could help to design peptides that selectively activate one pathway.

## 5. Discussion

In recent years, GPCR signaling has proved to be increasingly complex, and our understanding has changed from the simple inactive and active conformations to a much more diverse conformational landscape in which several conformations can differentially bind transducer proteins and trigger varied pathway activations. In the living organism, our understanding is now that GPCR binders are likely to trigger several pathways but at a different level of expression, depending on binders, GPCR localizations, expression levels, and others. It means that understanding in depth and as accurately as possible the subtle differences resulting in the activation of different pathways under the stimulus of different ligands on the same receptor is urgently needed. This is motivated not only by our wish for a better fundamental understanding of biological signaling, but also by direct applications in health, with the promise of more effective drugs with less adverse effects [217]. In this regard, many efforts have been made to develop new experimental approaches that can track these subtle changes, but in silico approaches are also contributing increasingly.

Since the beginning of the 2020s, significant progress has been made in the accurate modeling of varied GPCR conformations, thanks to the progress of deep learning approaches applied to protein structure modeling. However, if the perspective of systematic accurate modeling of the bound and unbound of GPCRs is clearly open, progress is still needed to access more specific conformational states underlying GPCR-biased signaling. Ways to rationalize their modeling are still missing, and particularly the number of experimentally resolved structures specific to a biased signaling currently available remains limited.

As discussed above, molecular simulations can contribute to identifying such conformations. Some rules seem to emerge particularly in terms of structural rearrangements of the 7 transmembrane helix bundle that lead to the activation of the G-protein or β-arrestin pathways. However, the amount of available simulation data still has to increase to be able to derive general tendencies underlying bias. Most simulations reported so far have been performed for class A GPCRs, the GPCR class associated with the shortest loops, and one can expect a valuable outcome from future simulations related to more GPCRs, particularly from other classes. Other directions that have been underinvestigated so far include GPCR multimerization, the role of lipids, and the impact of phosphorylations.

Another means of deciphering GPCR-biased signaling lies in the identification of biased drugs that are capable of specifically activating only one downstream pathway and thus easing the identification of the relevant conformations associated. Cheminformatic approaches (ligand-based or structure-based) combined with machine learning models have started to be used to predict ligand bias but are still far from routine usage. Particularly, it will require more data and applying the best practices to have an impact in this area. Finally, biased ligands are now seen as an opportunity to design new drugs with a very selective effect. The design process of this new generation of drugs must be considered in the light of recent developments in generative AI. The latest generative models including diffusion are particularly promising and should be properly combined with more traditional but proven approaches.

While our main focus here is to review computational approaches to study biased signaling, one cannot ignore the importance of advances in experimental techniques for the development of in silico tools. They are crucial both for validating these methods experimentally and for providing data to feed in silico learning. Unlike X-ray crystallography, which requires extensive sample preparation (thermostabilization, for example [218]), NMR can directly solve the structure of a wild-type GPCR [219], and chemical shifts measured with NMR can provide valuable information related to the local environment of a site of interest. ^19^F NMR has been successfully applied to explore biased signaling. It requires a ^19^F-containing probe that will bind to a cysteine residue, but it offers interesting benefits such as natural abundance, absence in GPCRs (resulting in no noise), and high sensitivity [220]. Using this approach, Liu and co-workers [221] have shown that they can distinguish between G-protein and β-arrestin bias in β2AR based on specific conformations of helices 6 and 7 monitored with ^19^F NMR. Research is ongoing to expand its applications with the design of new probes with increased conformational sensitivity [222] or probes that target other residues such as tyrosine [223]. Another approach is mass spectrometry (MS), which includes many powerful variants that can be used to investigate biased signaling. For example, limited proteolysis mass spectrometry (LiP-MS, a suitable MS technique to probe protein conformation changes based on signal difference between two samples undergoing distinct enzymatic digestion [224]) has recently been used to study the conformational dynamics of the adenosine receptor type 2A upon binding by various ligands [225]. Likewise, hydrogen–deuterium exchange mass spectrometry (HDX-MS) helped identify ICL3 mobility changes in a turkey β1-adrenergic receptor induced by the binding of biased ligands due to the distinct uptake of deuterium measured for this domain [226]. Finally, FRET and BRET could be used to monitor the dissociation of G-protein subunits, their recruitment to GPCR, their interaction with GRKs, the recruitment of β-arrestin by a GPCR, and more [227,228]. Given that these events are closely related to biased signaling, the data generated by these experiments could be exploited to train machine learning models to predict ligand bias as discussed in this review. The growing range of suitable experimental techniques presents an opportunity to properly validate computational predictions and combine them together to understand the complexity of biased signaling in depth. In particular, the insights from experimental studies could be used in conjunction with MD simulations to refine the structural observations and strengthen the findings.

Overall, GPCR-biased activation deciphering is most probably still in its infancy, and in the coming years, progress in both experimental and computational approaches should bring significant contributions. As for computational approaches, it is striking that deep learning approaches are now in all parts of the domain, even assisting molecular simulations, and it is highly probable that generative approaches will bring a significant contribution to bridging the gaps in our current understanding of biased signaling.

## Data Availability

No new data were created or analyzed in this study.
